# Dioscin attenuates D‑galactose‑induced retinal pigment epithelium senescence and epithelial–mesenchymal transition via the TGF‑β2/PI3K/Akt/β‑catenin pathway

**DOI:** 10.1371/journal.pone.0359423

**Published:** 2026-09-28

**Authors:** Qi Ouyang, Huiqin Zhou, Chenhao Ji, Hong Jiang, Linhui Zhao, Yiwei Pan, Shuanglin Xiang, Xiang Hu

**Affiliations:** 1 The National and Local Joint Engineering Laboratory of Animal Peptide Drug Development, College of Life Sciences, Hunan Normal University, Changsha, China; 2 Engineering Research Center for Antibodies from Experimental Animals of Hunan Province, College of Life Sciences, Hunan Normal University, Changsha, China; Tokyo University of Agriculture, JAPAN

## Abstract

**Purpose:**

This study aimed to evaluate the protective effects of Dioscin in an age-related macular degeneration (AMD) -like model and to explore its potential therapeutic mechanisms.

**Methods:**

The therapeutic potential of Dioscin for AMD was investigated using D-galactose-induced senescence models in ARPE-19 cells (an in vitro preliminary screening model) and C57BL/6J mice using RT-qPCR, western blotting, immunofluorescence, and SA-β-gal staining. Bioinformatic analysis and molecular docking identified TGF-β2 as a computationally predicted potential target of Dioscin in AMD. Key mechanisms involving TGF-β2 and the PI3K/Akt/β-catenin signaling pathway were assessed in vitro using RT-qPCR, western blotting.

**Results:**

In D-galactose-induced senescence models, Dioscin significantly alleviated RPE senescence and EMT. Single-cell transcriptome analysis revealed that TGF-β2 is highly expressed in choroidal vascular endothelial cells of AMD patients. Mechanistically, computational predictions (molecular docking and dynamics simulations) identified TGF-β2 as a computationally predicted potential target of Dioscin, and experimental data showed that Dioscin suppressed D-galactose-induced activation of the TGF-β2/PI3K/Akt/β-catenin pathway in RPE cells, suggesting a potential paracrine regulatory mechanism.

**Conclusion:**

Dioscin alleviates RPE senescence and EMT through suppression of the TGF-β2/PI3K/Akt/β-catenin pathway. Single-cell transcriptomics reveals that TGF-β2 is specifically upregulated in choroidal vascular endothelial cells in AMD, suggesting a paracrine mechanism linking vascular pathology to RPE dysfunction. These findings suggest that Dioscin may have potential value in mitigating RPE senescence and EMT in an AMD‑like model, although further validation in more clinically relevant AMD models is required.

## 1. Introduction

Age-related macular degeneration (AMD) is a leading cause of irreversible vision loss in the elderly, characterized by progressive dysfunction and degeneration of the retinal pigment epithelium (RPE) [1,2]. The early stages of dry AMD are primarily driven by RPE cellular senescence and epithelial-mesenchymal transition (EMT), yet effective pharmacological interventions targeting these core pathological processes remain limited [3]. Together, these factors represent a dual burden on both healthcare infrastructure and individual well-being.

AMD is clinically categorized into two main forms: the more common geographic atrophy (GA) form and the less common but more aggressive exudative form [4]. The pathogenesis of both forms is centered on the functional impairment and degeneration of the retinal pigment epithelium (RPE), a crucial cell layer that supports the health of photoreceptors [5]. The functional integrity of the RPE is crucial for maintaining retinal homeostasis, and RPE dysfunction represents a core element in the pathogenesis of AMD. In the early stages of AMD, RPE cells already exhibit functional abnormalities, including lipofuscin accumulation, impaired phagocytic capacity, and oxidative stress damage, which lay the groundwork for subsequent pathological changes [6]. In the GA form, RPE cells progress from early dysfunction to eventual atrophy and death, leading to secondary degeneration of overlying photoreceptors and the formation of map-like atrophic areas [7]. In the exudative form, compromised RPE barrier function triggers abnormal choroidal neovascularization, resulting in hemorrhage and fluid leakage [8]. Therefore, protecting and restoring RPE function has emerged as a critical target for the development of AMD therapeutic strategies.

The current therapeutic arsenal for AMD includes several pharmacological strategies. Intravitreal injection of anti-vascular endothelial growth factor (anti-VEGF) agents represents a foundational approach, effectively inhibiting pathological blood vessel growth and leakage to stabilize or improve visual acuity [9]. Combination therapy incorporating corticosteroid implants has shown utility in cases with suboptimal response to anti-VEGF monotherapy, particularly in resolving persistent retinal fluid [10]. Nutritional supplementation based on the AREDS and AREDS2 formulations provides a general risk-reduction strategy by supplying specific antioxidants and minerals [11]. More recently, targeted inhibition of the complement C3 pathway has demonstrated efficacy in slowing the progression of degenerative lesions, marking a shift toward mechanism-based intervention [12]. Despite these advances, limitations in functional benefits and long-term efficacy underscore the continued need for novel agents with diverse mechanisms of action, positioning natural compounds as a promising direction for future therapeutic development.

Dioscin is a naturally occurring steroidal saponin, found primarily in medicinal plants like *Paris polyphylla* and *Polygonatumspecies*, which exhibits a wide range of pharmacological activities, such as anti-tumor and anti-inflammatory effects [13]. Accumulating evidence has revealed the therapeutic potential of the Dioscin against various age-related diseases, largely attributed to its potent antioxidant, anti-inflammatory, and anti-senescence properties [14,15]. Specifically, Dioscin alleviates oxidative stress by inhibiting NOX2 to reduce ROS production and enhancing the activities of key antioxidant enzymes, such as SOD and GPx [16]. In a myocardial ischemia/reperfusion (MI/R) model, this protective effect was mediated through the activation of the Nrf2/HO-1 pathway [17]. Dioscin suppresses chronic inflammation (inflamm-aging) by inhibiting key pro-inflammatory signaling pathways, including NF-κB and AP-1, leading to reduced levels of cytokines such as TNF-α, IL-1β, and IL-6. In an Alzheimer’s disease model, Dioscin alleviated Aβ deposition and neuroinflammation, thereby improving cognitive function, via modulation of the RAGE/NOX4 pathway [18]. Accumulating evidence has shown that dioscin exerts anti‑senescence and anti‑EMT effects in various aging‑related models, including the aging brain [19]. While the pathogenesis of AMD similarly hinges on chronic oxidative stress, inflammation, and RPE senescence, processes that Dioscin is known to target, its potential efficacy in AMD remains strikingly unexplored. Therefore, investigating whether Dioscin can mitigate RPE degeneration in an AMD-like context represents a significant and promising research frontier.

Currently, the regulatory crosstalk between the choroidal vascular microenvironment and RPE cells in AMD remains incompletely understood, and the role of TGF-β2 in this intercellular communication has not been fully elucidated. In this study, we employed D-galactose-induced aging models in vivo and in vitro to evaluate the protective effects of Dioscin on RPE senescence and EMT. Through integrated bioinformatics analysis, molecular docking, and experimental validation, we investigated whether TGF-β2 serves as a molecular link connecting choroidal vascular pathology to RPE dysfunction and whether Dioscin exerts its effects by targeting the TGF-β2/PI3K/Akt/β-catenin signaling axis.

## 2. Materials and methods

### 2.1 Chemical and reagents

D-galactose (D-gal), Methylthiazolyldiphenyl-tetrazolium bromide (MTT) and Crystal violet were purchased from Sangon (Shanghai, China) and Dioscin was obtained from Macklin (Shanghai, China). Vitamin D3 (VitD3) was purchased from Coolaber (Beijing, China). SA-β-Gal Staining Kit was obtained from Beyotime (Shanghai, China). Primary antibodies: p21, p16, p-PKC (Selleck, Shanghai, China); MMP1, Goat Anti-Rabbit IgG H&L (HRP) (Zenbio, Chengdu, China); E-cadherin, N-cadherin, PI3K, Akt, p-PI3K, p-Akt, PKC, β-catenin, GSK-3β (Immunoway, Texas, USA). β-actin, GAPDH, DAPI, Alexa Fluor® 488-conjugated Goat Anti-Rabbit IgG, Cy3 conjugated Goat Anti-mouse IgG (H + L) (Servicebio, Wuhan, China).

### 2.2 Cell culture and treatment

The human retinal epithelial cells (ARPE-19, Zhong Qiao Xin Zhou Biotechnology, Shanghai, China) were cultured in DMEM/F12 medium contain 10% FBS (Gibco, USA),100U/mL penicillin and 100 ug/mL streptomycin at 37 ℃,5% CO_2_. This study used human retinal pigment epithelial cell line ARPE-19 for preliminary in vitro experiments. This cell line is a classic cell model for screening anti RPE cell aging and EMT inhibitors in vitro, and can achieve rapid in vitro validation of drug activity. This study used ARPE-19 cells in a sub fused state, focusing on the regulatory effects of drugs on the aging and EMT phenotype of RPE cells. ARPE-19 cells were treated with D-gal (2 mM) for 48 h to induce cellular senescence. Dioscin (2 μM) and Vitamin D3 (VitD3, 20 μM) were co-applied during the same 48 h period to evaluate their protective effects against D-gal-induced senescence.

### 2.3 Animals and treatment

Male C57BL/6J mice (6 weeks, 18−20 g, Hunan SJA Laboratory Animal Co., Ltd, China) were used. All experimental procedures were approved by the Animal Ethics Committee of Hunan Normal University (2024−776). The mice were maintained under standard housing conditions with a 12-hour light/dark cycle, controlled temperature and humidity, and were provided ad libitum access to standard laboratory diet and water.

This model simulates age‑related RPE cell aging, EMT, and retinal structural disorder, representing an AMD‑like RPE senescence model. The mice were randomly divided into D-gal induced group for aging model and Dioscin treated group for delaying aging model. The experimental group was divided into four groups (*n* = 5): control group (Mock), Dio-treated group, D-gal-induced group, D-gal + Dio-treated group (D-gal, 200 mg/kg, once per day, intraperitoneal injection; Dioscin, 80 mg/kg, every 2 days, gavage injection) [20,21].

### 2.4 Animal monitoring and humane endpoints

Body weight was recorded daily at the same time point. Mice were monitored daily for signs of distress, including hunched posture, ruffled fur, > 20% body weight loss, or impaired mobility. These signs were pre-defined as humane endpoints for early euthanasia. No animals died or required euthanasia prior to the scheduled experimental endpoint. The animal study was reviewed and approved by the Institutional Animal Care and Use Committee (IACUC), Hunan Normal University (2024−776).

### 2.5 Euthanasia and tissue collection

After 13 weeks, all mice were anesthetized with carbon dioxide (CO_2_) using a gradual fill method. While under deep anesthesia, mice were euthanized by exsanguination via retro-orbital blood collection, consistent with AVMA guidelines. Eyeballs were subsequently collected for further experiments.

### 2.6 Quantitative reverse transcription polymerase chain reaction (RT-qPCR)

Total RNA was isolated from retina tissues, RPE/choroid or ARPE19 cells with TRIzol reagent (Takara, Beijing, China) following the manufacturer’s protocol. Equal amounts of RNA were reverse transcribed into cDNA using HiScript® II Q RT SuperMix (YoungGen, Kunming, China). Quantitative real-time PCR was then carried out on a Bio-Rad CFX Real-Time PCR System with SYBR Green qPCR Master Mix (YoungGen, Kunming, China). Gene expression levels were normalized to *β-actin* and analyzed via the 2^−ΔΔCT^ method. All primer sequences used in this study are listed in S1 Table.

### 2.7 Hematoxylin and eosin (H&E) staining

For hematoxylin and eosin (H&E) staining, retinal tissue sections (4 μm thick) embedded in paraffin were subjected to dehydration through a series of ethanol solutions with increasing concentrations (75%, 85% and 100%). Staining was performed using a commercial H&E staining kit (GP1031, Servicebio, Wuhan, China). The stained sections were observed and imaged under a light microscope (Olympus, Japan) at 4× and 20 × magnification.

### 2.8 Immunofluorescence (IF)

The immunofluorescence (IF) staining protocol was performed on cells or paraffin sections as follows: fixation (Fixative Solution, 15 min), permeabilization (0.1% Triton X-100, 30 min), and blocking (5% BSA, 1 h). Samples were then incubated overnight at 4°C with a panel of primary antibodies—including p21 (1:100), p16 (1:100), Vimentin (1:200), E-cadherin (1:100), N-cadherin (1:100) -followed by a 2-hour incubation with an Alexa Fluor® 488-conjugated secondary antibody or Cy3 conjugated secondary antibody (1:500, Servicebio, Wuhan, China). After nuclear staining with DAPI (10 min), imaging was carried out on a laser scanning confocal microscope (EVOS M5000, Thermo Fisher, Waltham, MA, USA) at 20 × magnification.

### 2.9 Western Blotting

Proteins were extracted using RIPA buffer (CWBIO, China). 10 μg of protein per sample were separated by SDS−PAGE, transferred to PVDF membranes, and incubated with primary antibodies overnight at 4 °C. After incubation with HRP−conjugated secondary antibodies, bands were visualized using an ECL system (Suzhou, China). Quantitative densitometric analysis was performed on the immunoreactive bands using ImageJ software v 1.52a (National Institutes of Health, USA). To account for variations in protein loading, the optical density of each target protein band was normalized to that of the internal control (β−actin) within the same sample. Statistical analysis was conducted on data from at least three independent replicates.

### 2.10 Senescence-associated-β-galactosidase (SA-β-gal) assay

ARPE-19 cells and the frozen sections of mice were stained with the SA-β-Gal Staining Kit (C0602, Beyotime). Briefly, cultured cells and frozen sections were fixed for 15 min and then incubated with SA-β-gal staining reagent at 37 °C overnight. The images of stained-positive cells were counted under light microscopy (20 × , Olympus, Japan). The percentage of senescent cells was determined by the ratio of blue (SA-β-Gal-positive) cells to total cells obtained from 5 random view fields of each sample.

### 2.11 Acquisition and analysis of bulk and single-cell RNA-seq data

The macular region of the RPE-choroid complex, which is responsible for high-acuity vision, is the primary target of pathology in AMD. Due to its high metabolic demand and critical function, this region is particularly vulnerable, playing a central role in the disease’s pathogenesis [22]. Therefore, to investigate the molecular mechanisms underlying AMD, we analyzed 91 transcriptome samples from the GSE29801 dataset in the Gene Expression Omnibus (GEO) database, which included 50 normal and 41 AMD macular RPE-choroid samples [23]. The transcriptome data were normalized, and differential expression analysis was conducted using the “limma” package in R. The expression data were log-transformed, and genes with an adjusted p-value < 0.05 and an absolute log_2_ fold change (|log_2_FC|) > 0.5 were defined as differentially expressed genes (DEGs) associated with AMD [24]. The volcano plot was generated using the “ggplot2” package for visualization. We further obtained and analyzed single-cell RNA sequencing data of the macular choroid from the GSE183320 dataset, which includes 10 healthy donors and 11 patients with AMD [25]. Data processing was carried out using the Seurat package in R. The “NormalizeData” function was applied for normalization, and cell clustering was performed using the “FindClusters” function at a selected resolution. Dimensionality reduction and visualization were conducted via the “RunUMAP” function.

### 2.12 Gene set enrichment and Protein-Protein Interaction (PPI) network analysis

To evaluate the results of the differential gene expression analysis, we performed Gene Ontology (GO) enrichment analysis, categorizing terms into biological processes (BP), cellular components (CC), and molecular functions (MF), using the “clusterProfiler” package in R [26]. This analysis preliminarily elucidated the potential biological functions of the differentially expressed genes. To further identify core genes associated with AMD, a protein-protein interaction (PPI) network was constructed using the online STRING database [27] and visualized with Cytoscape 3.10.1 [28]. By calculating the degree value of each node in the network and subsequently applying the MCODE algorithm [29] to extract key functional modules with the parameters set as follows: Degree Cutoff = 2, Node Density Cutoff = 0.1, Node Score Cutoff = 0.1, K-Core = 2, and Max. Depth = 100, we identified potential functional modules and candidate genes that may play central roles in the pathogenesis of AMD.

### 2.13 Prediction of potential AMD targets for dioscin

PharmMapper (http://lilab-ecust.cn/pharmmapper/) is an online server designed for potential drug target identification using a pharmacophore mapping approach. It employs statistical methods to match small molecules against a comprehensive database of pharmacophore models derived from known protein targets [30]. In this study, the 2D structure of Dioscin (PubChem CID: 119245) was retrieved from PubChem [31] and uploaded to the PharmMapper server. Reverse docking was performed using the “Human Protein Targets Only” filter, while other parameters were kept as default. This process identified potential protein targets of Dioscin. Subsequently, the GeneCards database (https://www.genecards.org/) was queried using the keyword “Age-related Macular Degeneration” to obtain genes strongly associated with AMD (Relevance score > 20) [32]. These genes were then intersected with the core functional module genes obtained from a PPI network analysis. The overlapping genes were considered as potential AMD-related targets of Dioscin.

### 2.14 Molecular docking

To elucidate the binding mechanism between Dioscin and the predicted core targets, molecular docking simulations were performed. The 2D structure of Dioscin was first converted into a 3D structure using Chem3D Ultra 14.0 and subsequently energy-minimized with the MM2 algorithm to obtain a stable low-energy conformation [33]. The crystal structures of the selected core proteins were retrieved from the Protein Data Bank (https://www.rcsb.org) [34]. These target protein receptors were preprocessed in PyMOL by removing water molecules and organic ligands. Using AutoDockTools 1.5.7, hydrogen atoms were added, and Gasteiger charges were calculated for the receptors. Both the Dioscin and the target proteins were then converted into the PDBQT format. Appropriate grid box parameters were defined to encompass the binding site of each target. Finally, the docking calculation was executed with AutoDock Vina 1.1.2 to evaluate the binding affinity, expressed as docking energy scores. The resulting binding poses were visualized using PyMOL to identify the potential amino acid residues involved in Dioscin binding [35,36].

### 2.15 Molecular dynamics

Molecular dynamics (MD) simulations of the TGFβ2-Dioscin complex were executed using GROMACS 2020.3. The protein and ligand were parameterized using the Amber ff14SB and GAFF force fields, respectively, with ligand topologies generated via AmberTools and ACPYPE. The complex was solvated with the SPC216 water model in a periodic truncated octahedron box, maintaining a 1.2 nm buffer distance, and neutralized with Na^+^ and Cl^-^ ions.

The system underwent steepest-descent energy minimization (50,000 steps), followed by two successive 100 ps cycles of NVT and NPT equilibrations with heavy-atom position restraints. The v-rescale thermostat (300 K) and Parrinello-Rahman barostat (1.0 bar) were applied for temperature and pressure controls, respectively. Finally, a 50 ns unrestrained production MD simulation was conducted with a 2 fs time step. All hydrogen-involved bonds were constrained using the LINCS algorithm, and long-range electrostatics were treated via the Particle Mesh Ewald (PME) method. Trajectories were sampled every 10 ps, and subsequent structural properties were analyzed using GROMACS utilities and plotted in R studio.

### 2.16 Statistical analysis

All experimental data were obtained from at least three independent replicates. For animal experiments, mice were randomly assigned to treatment groups, and outcome assessments (including histology, immunofluorescence, and qPCR) were performed blinded. Sample size (n = 5 per group for in vivo studies; n = 3 per group for in vitro studies) was determined based on power analysis from preliminary experiments. ImageJ software v 1.52a (National Institutes of Health, USA) was employed for data quantification. Subsequent statistical analysis and graph generation were performed using GraphPad Prism 8.0. Before applying parametric tests, the normality of data distribution was assessed using the Shapiro–Wilk test. Data that passed normality and homogeneity of variance were analyzed using Student’s two-tailed t-test (for two groups) or one-way ANOVA with Tukey’s post-hoc test (for multiple groups). For data that did not meet normality assumptions, the non-parametric Mann–Whitney U test or Kruskal–Wallis test was applied accordingly. Results are expressed as mean ± SD, and statistical significance was set at p < 0.05.

## 3. Results

### 3.1 Dioscin ameliorates D-gal-induced senescence in C57 mice

To investigate the role of Dioscin in senescence, Dioscin was used to D-gal-induced aging mice. As shown in Fig 1A, the mice were monitored throughout the modeling process. Body weight of mice was significantly decreased by D-gal (p < 0.01). Dioscin restored D-gal-induced changes in weight (Fig 1B). H&E staining revealed that D-gal treatment caused significant disorganization of the RPE layer, including irregular cell arrangement, loss of monolayer integrity, and focal RPE thinning (Fig 1C, arrows). These morphological changes are characteristic features of retinal aging and have been associated with impaired RPE function, reduced photoreceptor support, and increased susceptibility to AMD-like degeneration. Notably, Dioscin co-treatment markedly preserved RPE monolayer architecture, indicating its protective effect against D-gal-induced retinal senescence. Aβ was senescence biomarker as described previously. The number of SA-β-gal positive cells was significantly increased in the D-gal-treated group, while Dioscin treatment reversed this phenomenon (Fig 1D-1E). For additional molecular validation, we examined the mRNA expression of key senescence-associated genes, *p16* and *p21*. The results showed that the transcript levels of both *p16* and *p21* were markedly up-regulated in the RPE and retinal tissues of the D-gal-treated group, and these increases were significantly suppressed by Dioscin treatment (Fig 1F-1G). Consistent with these data, immunofluorescence staining of retinal sections also revealed a pronounced elevation of p16 protein expression following D-gal induction, which was likewise attenuated by Dioscin (Fig 1H-1I).

**Fig 1:**
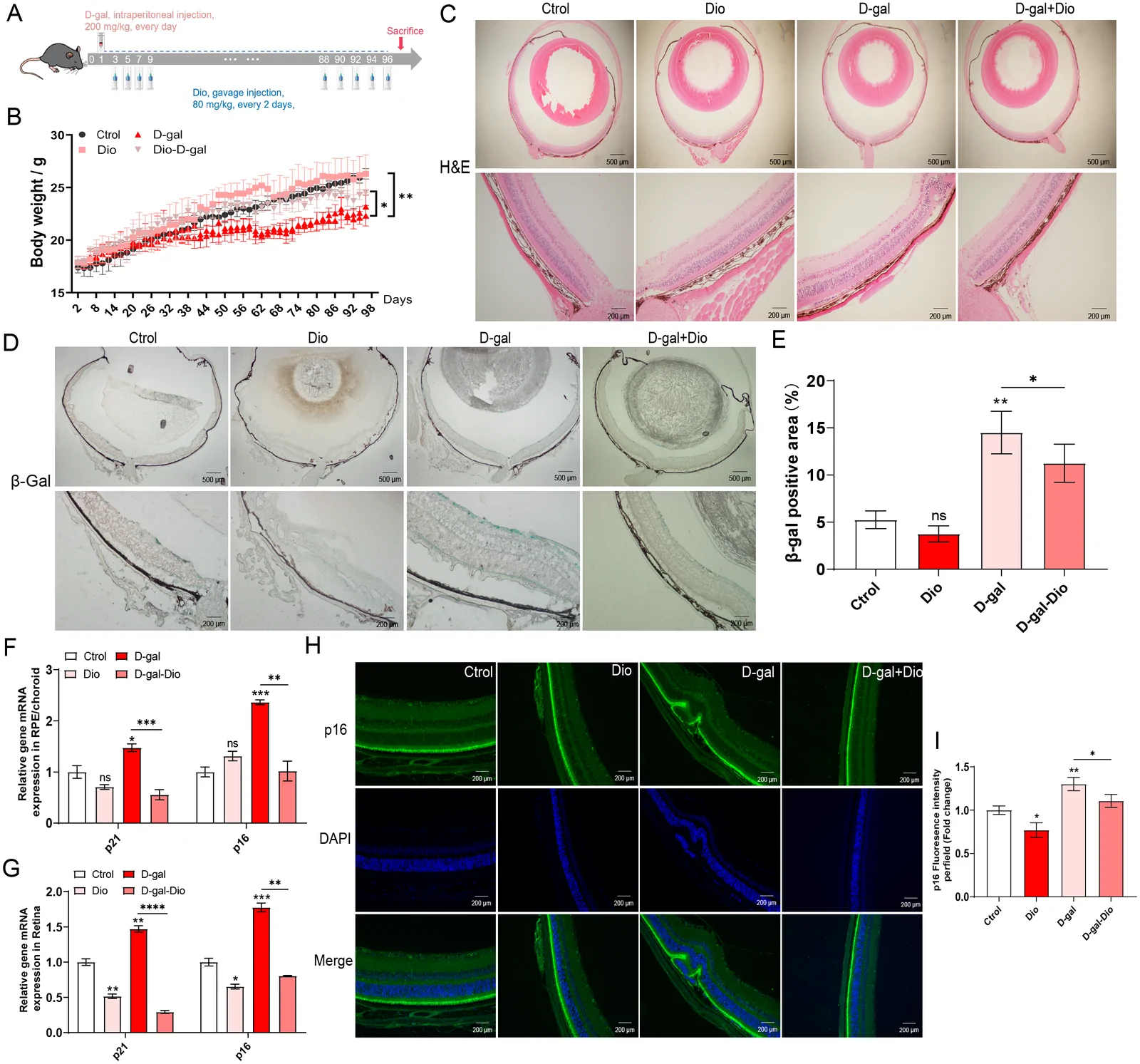
Dioscin ameliorates D-gal-induced senescence in C57 mice. (A) Experiment timeline of Dioscin and D-gal treatment in C57 mice. (B) Body weight of various treatment groups (*n* = 5 mice per group). (C) H&E staining of mouse retina (*n* = 5 mice per group). The yellow arrow indicates displaced nuclei originating from the inner nuclear layer, which are ectopically located within the RPE/choroid (pigmented layer). the red arrow points to the retinal pigment epithelium (RPE) and choroid. Scale bar, 500 μm/200 μm. (D-E) SA-β-gal staining of mouse retina (*n* = 5 mice per group). (F) Relative mRNA levels of *p21* and *p16* in mouse RPE/choroid (*n* = 3 independent experiments). (G) Relative mRNA levels of *p21* and *p16* in mouse retina (*n* = 3 independent experiments). **(H-I)** Protein expression of p16 in mouse retina evaluated by immunofluorescence (Each data point represents the mean intensity from 5 random fields of view per sample). Scale bar, 200 μm. Data are shown as mean ± S.D. Statistical significance was determined by one-way ANOVA with Tukey’s post-hoc test. *p < 0.05, **p < 0.01, ***p < 0.001, ****p < 0.0001; ns, not significant.

These results suggest that Dioscin may have potential therapeutic value for AMD by mitigating RPE senescence and EMT, warranting further investigation in more clinically relevant AMD models.

### 3.2 Dioscin influenced epithelial-mesenchymal Transition in C57 mice

Given the role of epithelial-mesenchymal transition (EMT) in AMD pathogenesis, we evaluated whether Dioscin modulates this process in the RPE of D-gal-induced aging mice by assessing the expression of Vimentin, N-cadherin and E-cadherin.

Immunofluorescence staining revealed that D-gal induction triggered a pronounced EMT phenotype, characterized by a significant upregulation of Vimentin and downregulation of E-cadherin. Notably, Dioscin co-treatment effectively mitigated these changes, restoring E-cadherin expression and reducing Vimentin levels. Furthermore, Dioscin alone significantly suppressed basal Vimentin expression but did not affect E-cadherin (Fig 2A-2C). Consistent with these findings, qPCR analysis confirmed the regulatory trends at the transcriptional level for *Vimentin* and *N-cadherin*, two key mesenchymal markers. The mRNA expression levels of these mesenchymal markers were significantly increased upon D-gal induction, and this upregulation was markedly suppressed by Dioscin co-treatment (Fig 2D-2E).

**Fig 2:**
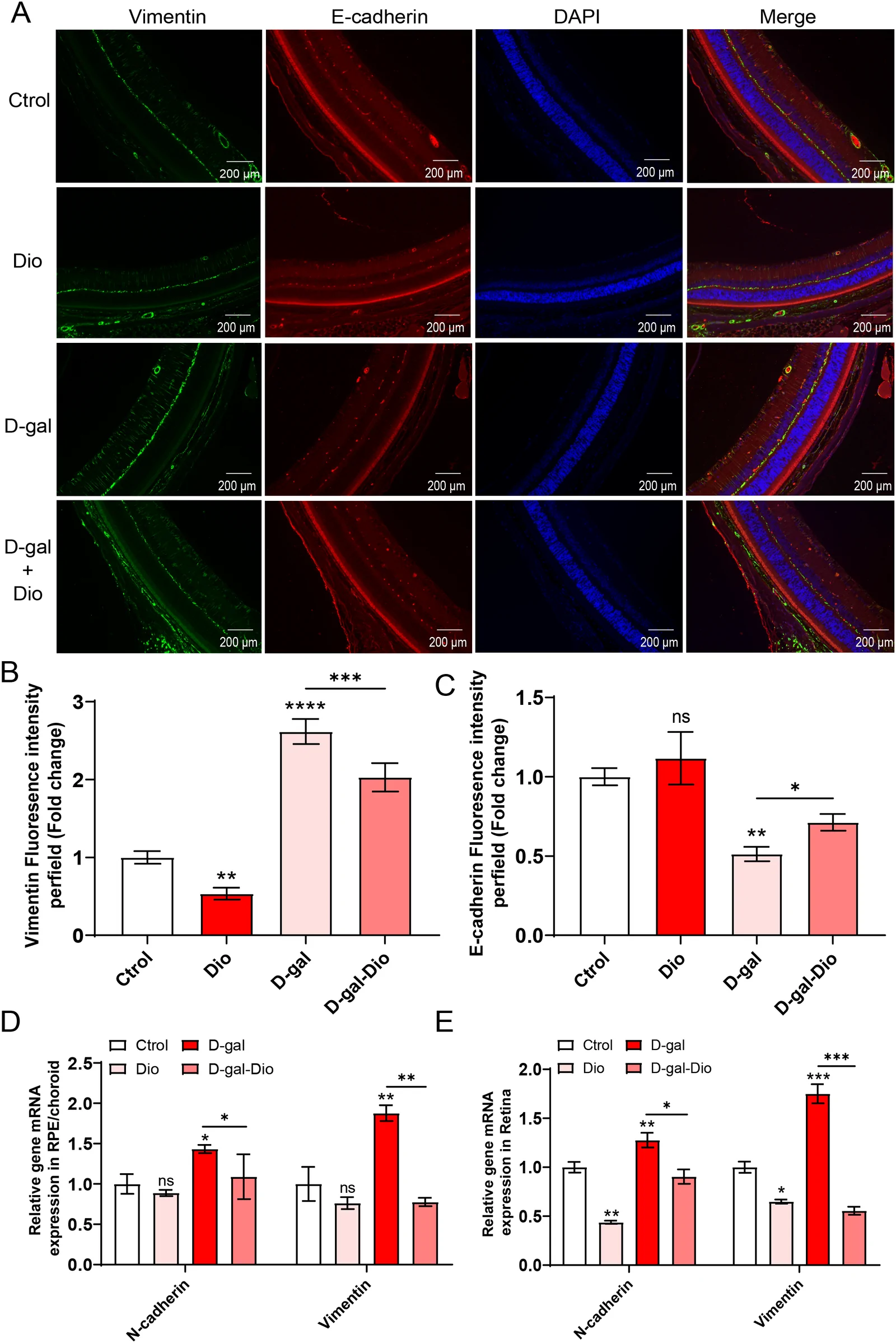
Dioscin attenuates epithelial-mesenchymal Transition in C57 mice. (A-C) The protein of Vimentin and E-cadherin in mouse retina was evaluated by immunofluorescence. Each data point represents the mean intensity from 5 random fields of view per sample. Scale bar, 200 μm. (D-E) The mRNA levels of *vimentin* and *n-cadherin* in mouse RPE/choroid and retina were evaluated by RT-qPCR (*n* = 3 independent experiments).

These results indicate that Dioscin attenuates D-gal-induced EMT in mouse RPE at the protein and transcriptional levels, supporting its potential role in maintaining RPE homeostasis.

### 3.3 Dioscin ameliorates D-gal induced senescence in ARPE-19 cells

D-galactose serves as a stable inducer of cellular senescence, primarily through the accumulation of metabolic byproducts that mimic natural aging. Hence, we established a senescent cell model of ARPE-19 using a low dose of D-gal in vitro. Initially, we determined the non-cytotoxic concentration ranges for Dioscin, D-gal, and Vitamin D3 (VitD3, a positive control) through cytotoxicity assays in ARPE-19 cells. The concentrations of D-gal (2 mM) and Dioscin (2 μM) were selected based on previous literature [37] and preliminary cytotoxicity assays. MTT assays confirmed that these concentrations were non‑cytotoxic to ARPE-19 cells (Fig 3A-3C). Therefore, subsequent experiments were conducted using 2 mM D-gal, 2 μM Dioscin, and 20 μM VitD3.The number of SA-β-gal-positive (blue) cells was significantly higher in the D-galactose-treated group compared to the control group, and this increase was markedly reversed by treatment with either Dioscin or VitD3 (Fig 3D-3E). Assessment of cellular senescence by immunofluorescence staining for p16 revealed that its expression was significantly elevated in D-galactose-induced ARPE-19 cells, an effect that was also reversed by Dioscin treatment (Fig 3F-3G). Furthermore, Western blot analysis demonstrated that the expression of senescence-associated proteins (p16, p21, MMP1) was significantly up-regulated in D-galactose-treated ARPE-19 cells, and this up-regulation was effectively suppressed by either Dioscin or VitD3 treatment (Fig 3H-3K).

**Fig 3:**
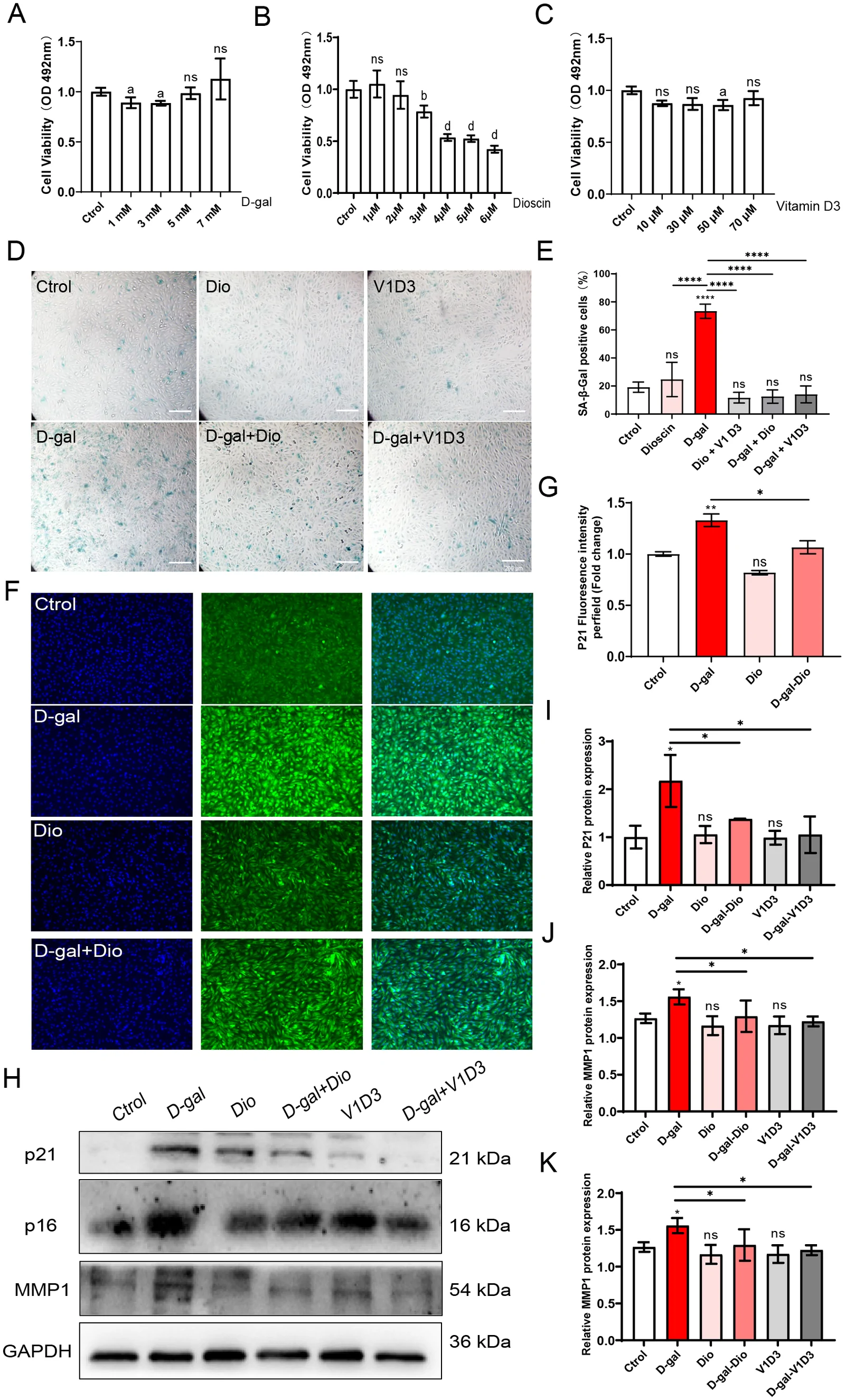
Dioscin ameliorates D-gal induced senescence in ARPE-19 cells (in vitro preliminary screening model). (A-C) Cell viability of ARPE-19 cells treated with indicated doses of D-gal, Dioscin, and Vitamin D3 (VitD3, positive control) determined by MTT assay (n = 3 independent experiments). (D-E) SA-β-gal staining of ARPE-19 cells (*n* = 3 independent experiments). (F-G) The protein of p16 in ARPE-19 cells was evaluated by immunofluorescence (Each data point represents the mean intensity from 5 random fields of view per sample). Scale bar, 200 μm. (H-K) The protein of p21, p16 and MMP1 in ARPE-19 cells was assessed by Western Blot (*n* = 3 independent experiments).

Taken together, these results demonstrate that D-gal successfully induces a senescent state in ARPE-19 cells, as evidenced by increased SA-β-gal activity and upregulation of key senescence markers (p16, p21, MMP1). Dioscin effectively mitigated this D-gal-induced senescence.

### 3.4 Dioscin influenced epithelial-mesenchymal Transition in ARPE-19 cells

Having established the anti-senescence effects of Dioscin, we next sought to investigate its potential role in modulating the EMT, a process closely associated with cellular senescence and AMD. To further investigate the anti-aging effect of Dioscin on migration and invasion, we performed wound-healing and transwell assays in ARPE-19 cells for 24 h after incubation with different treatment. Both wound healing and transwell assays demonstrated that D-gal treatment significantly accelerated cell migration and invasion, respectively. Conversely, Dioscin alone showed no intrinsic effect but effectively attenuated the pro-migratory and pro-invasive effects induced by D-gal (Fig 4A-4D). To elucidate the underlying mechanism behind the observed changes in cell migration and invasion, we examined the expression of key EMT markers (E-cadherin, N-cadherin, and Vimentin) at both transcriptional and protein levels. The results of EMT markers genes (*N-cadherin*, and *Vimentin*) significantly increased, *E-cadherin* decreased in response to D-gal exposure and were notably suppressed by Dioscin (Fig 4E-4G). Besides, our results indicated that Dioscin treatment reverses the upregulation of EMT biomarkers such as N-cadherin, and Vimentin under D-gal induced condition (Fig 4H-4I).

**Fig 4:**
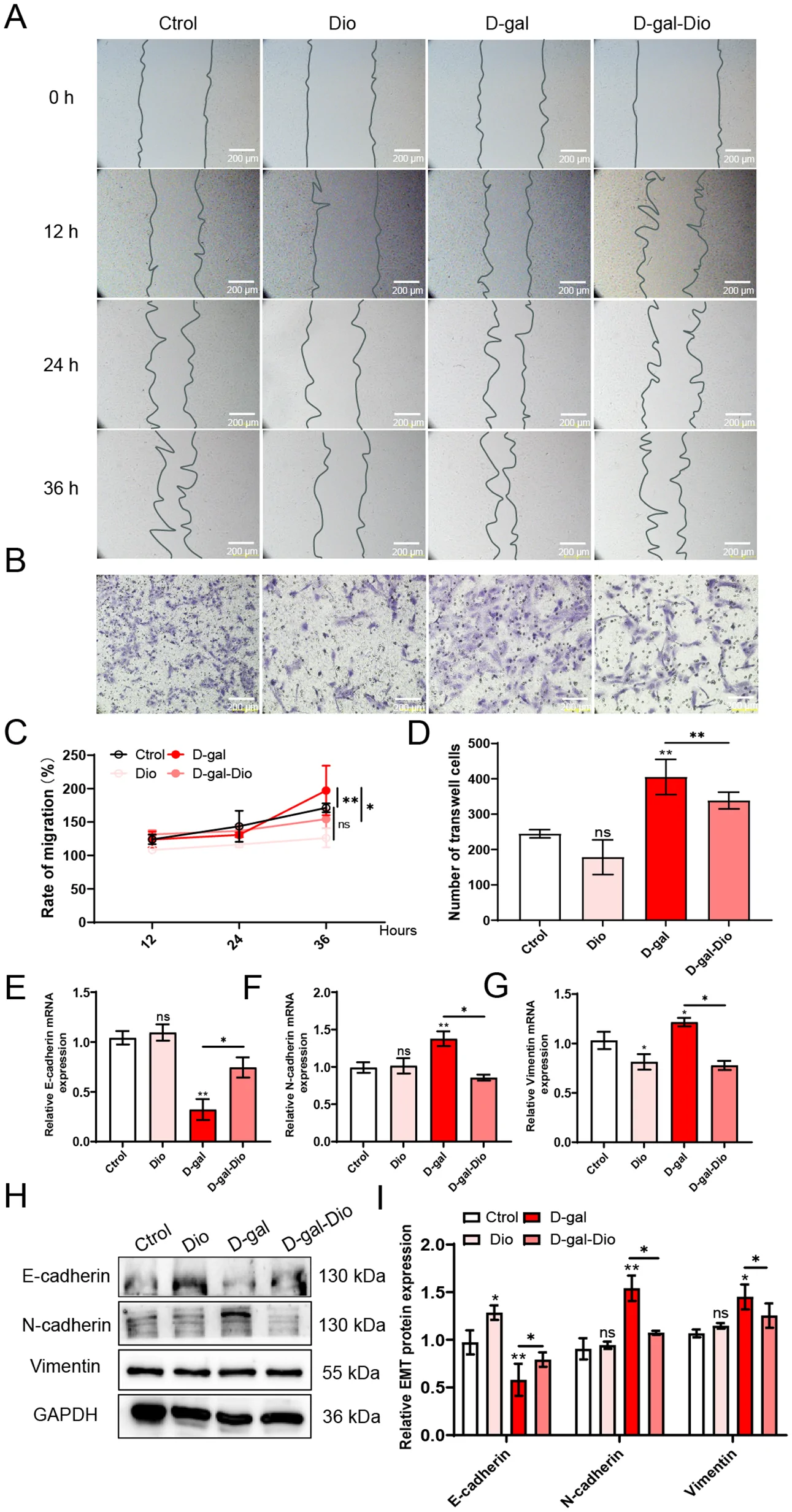
Dioscin suppresses epithelial-mesenchymal Transition in ARPE-19 cells (in vitro preliminary screening model). (A) Representative images of wound-healing assay showing ARPE-19 cell migration at 0 and 24 h (*n* = 3 independent experiments). (B) Representative images of Transwell invasion assay (*n* = 3 independent experiments). (C-D) Quantification of relative migration distance and invasion rate (*n* = 3 independent experiments). (E-G) Relative mRNA levels of *E-cadherin*, *N-cadherin* and *Vimentin* in ARPE-19 cells determined by RT-qPCR (*n* = 3 independent experiments). **(H-I)** Protein levels of E-cadherin, N-cadherin, and Vimentin assessed by Western blot (*n* = 3 independent experiments). Data are shown as mean ± S.D. Statistical significance was determined by one-way ANOVA with Tukey’s post-hoc test. *p < 0.05, **p < 0.01, ***p < 0.001, ****p < 0.0001; ns, not significant.

Taken together, these data indicate that Dioscin counteracts the D-gal-triggered enhancement of cell motility by modulating key EMT markers, thereby restoring the epithelial phenotype and revealing a novel mechanism underlying its anti-aging efficacy.

### 3.5 Bioinformatic analysis suggests TGF-β2 as a potential mediator linking Dioscin to AMD pathogenesis

To identify DEGs between AMD patients and normal controls, we analyzed gene expression data from the RPE of the macular region using the GSE29801 dataset. Differential expression analysis was conducted following data normalization. A total of 116 DEGs were identified, including 71 up-regulated and 45 down-regulated genes. The results are visualized in the volcano plot (Fig 5A). GO enrichment analysis revealed that these DEGs were significantly enriched in key pathways relevant to AMD pathogenesis. Notably, these included biological processes such as “response to vitamin” and “response to nutrient” highlighting metabolic disturbances; the cellular component “collagen-containing extracellular matrix”, implying impairments in the structural scaffold that supports retinal cells and regulates nutrient exchange; and molecular functions including “heparin binding” and “glycosaminoglycan binding”, suggestive of dysregulated growth factor signaling and inflammation (Fig 5B).

**Fig 5:**
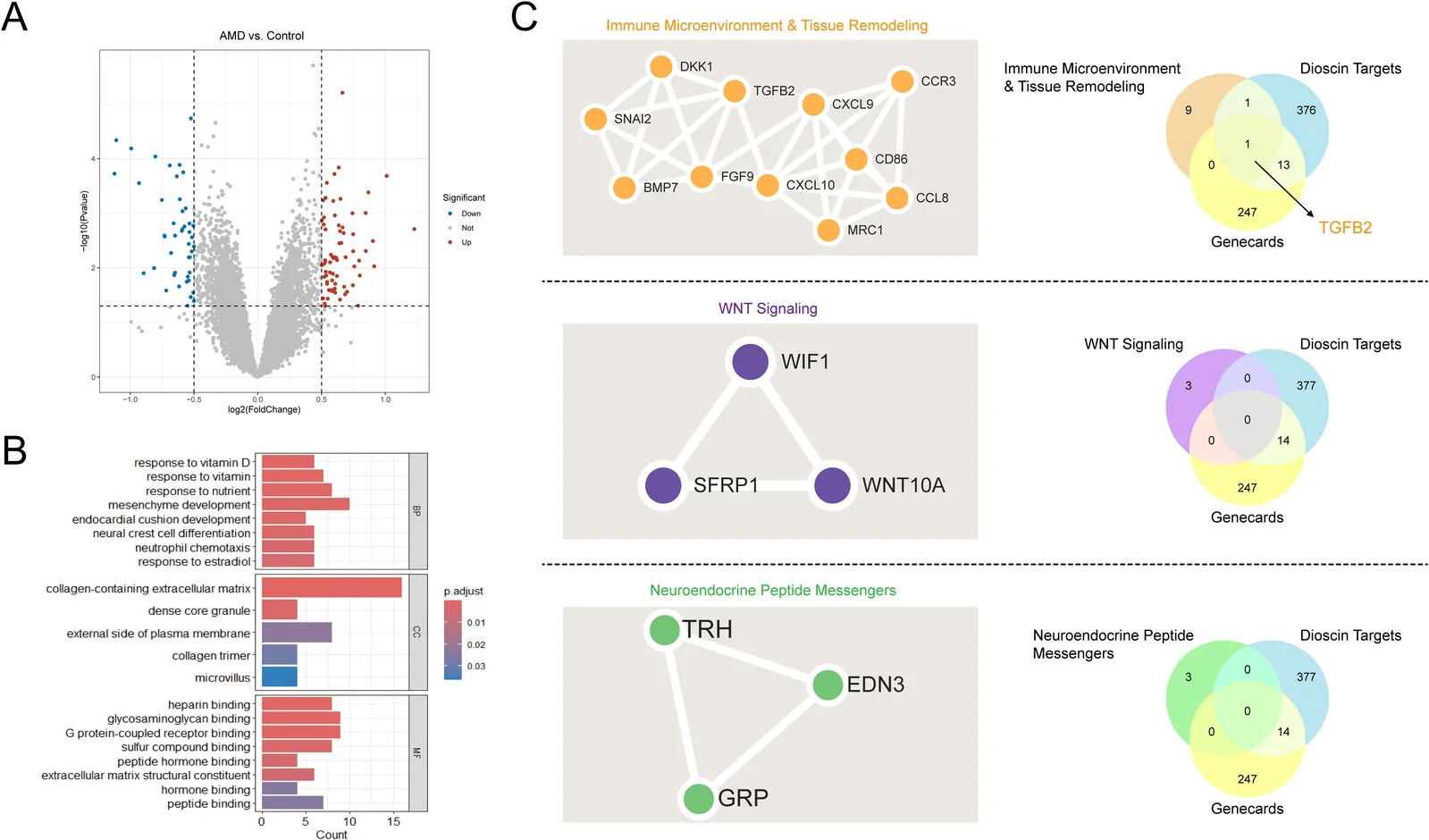
Bioinformatic screening identifies TGF-β2 as a potential mediator linking Dioscin to AMD pathology. (A) Volcano plot showing differentially expressed genes (DEGs) in macular RPE-choroid between AMD patients and normal controls from GSE29801 dataset. Red and blue dots indicate significantly upregulated and downregulated genes, respectively (|log_2_FC| > 0.5, adjusted p < 0.05). (B) Gene Ontology (GO) enrichment analysis of DEGs, showing top terms in Biological Process (BP), Molecular Function (MF), and Cellular Component (CC). (C) Multi-tiered screening strategy: intersection of key MCODE-derived PPI subnetworks from AMD-associated DEGs, known AMD-related genes from GeneCards database (relevance score > 20), and predicted Dioscin targets from PharmMapper analysis. TGF-β2 was identified within the “Immune Microenvironment & Tissue Remodeling” subnetwork.

To further explore the potential targets of Dioscin in the context of AMD, we first employed the PharmMapper server to predict its potential binding proteins, yielding a preliminary set of 391 targets. In parallel, a PPI network was constructed using the AMD-related DEGs. To identify densely connected functional modules that might represent core pathological mechanisms, we performed module analysis using the MCODE algorithm. This analysis revealed three principal subnetworks, which were functionally annotated as “Immune Microenvironment & Tissue Remodeling”, “WNT Signaling”, and “Neuroendocrine Peptide Messengers.” Finally, the molecular constituents of these three key subnetworks were intersected with both the predicted Dioscin targets and known AMD-associated genes from the GeneCards database. This multi-tiered screening approach identified TGF-β2 specifically within the “Immune Microenvironment & Tissue Remodeling” subnetwork, suggesting that Dioscin may influence AMD progression by targeting this molecule (Fig 5C). These findings provide a bioinformatic basis for hypothesizing that TGF-β2 may be involved in the therapeutic effects of Dioscin in AMD, which was subsequently explored in our experimental models.

### 3.6 Molecular docking and molecular dynamics identify TGF-β2 as a putative binding target of dioscin

To explore the potential interaction between Dioscin and the predicted target TGF-β2, we first performed a molecular docking simulation. The two-dimensional structure of Dioscin was converted into a three-dimensional format using the MM2 algorithm for energy minimization (Fig 6A). This optimized structure was then docked into the crystal structure of TGF-β2 (PDB ID: 6I9J) retrieved from the Protein Data Bank. The docking analysis revealed the binding mode of Dioscin within the pocket of TGF-β2 and its specific interactions with surrounding amino acid residues. Among the conformations generated, the three poses with the lowest binding energies were selected for in-depth analysis. Our results demonstrated a favorable binding energy for the Dioscin-TGF-β2 complex. Further analysis of the binding interface showed that Dioscin may form close interactions with ASN405, MET406, CYS350, SER377, SER404 and TYR341 residues of TGF-β2 (Fig 6B). To further evaluate the structural stability of the predicted complex, a 50 ns molecular dynamics (MD) simulation was performed. As shown in Fig 6C, the RMSD of TGF-β2 C-alpha atoms increased during the first 5 ns, reflecting initial structural adaptation, then stabilized from 10 ns onward within a range of 2.0–3.5 Å, indicating maintained structural integrity. RMSF analysis (Fig 6D) revealed that core domains (residues 310–350 and 375–390) exhibited low fluctuation (< 2.0 Å), suggesting rigidity in the putative Dioscin-binding pocket. A flexible loop region (residues 355–375) showed higher fluctuation (> 5.0 Å), which may reflect an induced-fit mechanism that optimizes binding. Together, these computational results indicate that TGF-β2 as a computationally predicted potential target of Dioscin.

**Fig 6:**
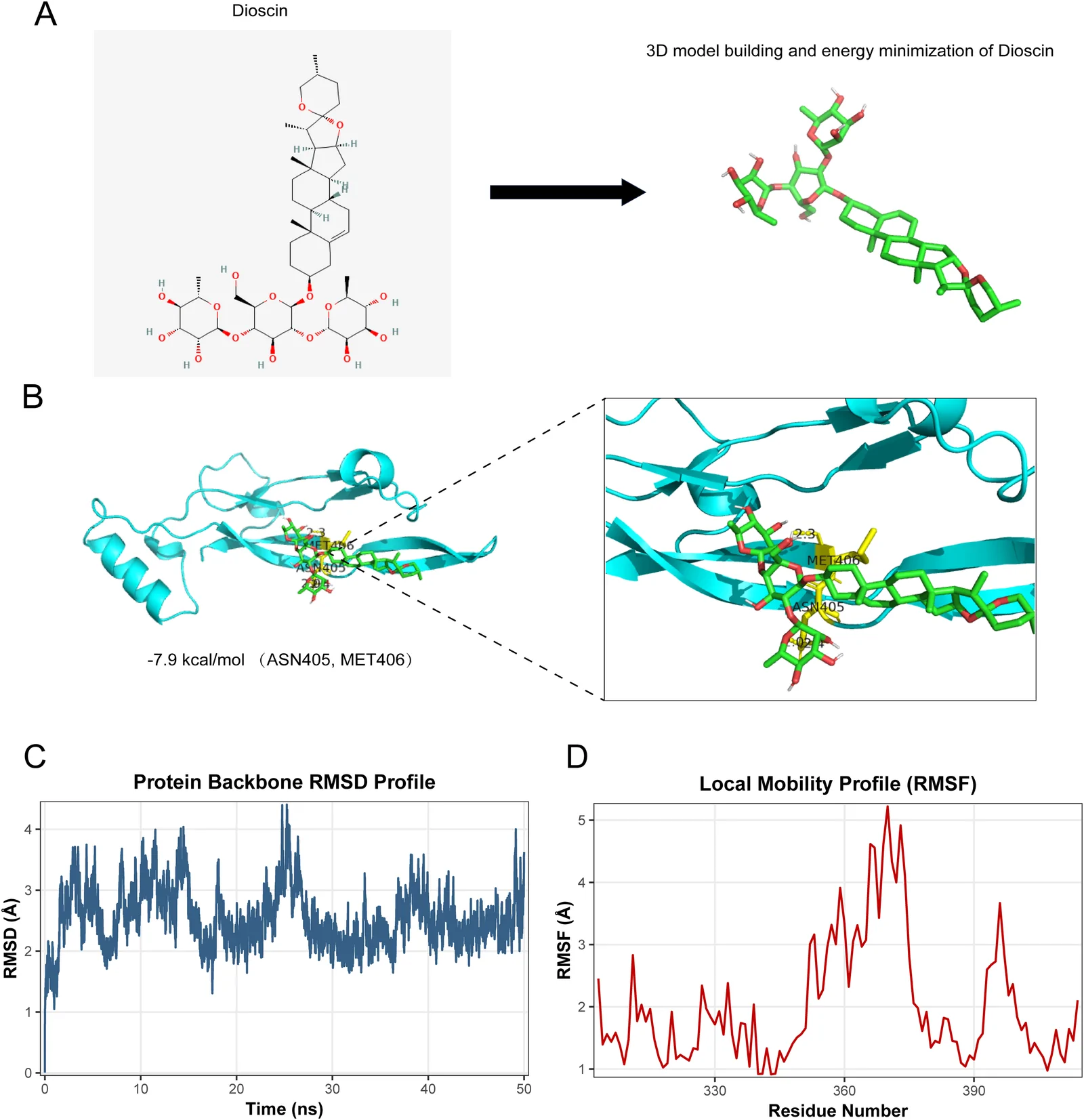
Molecular Docking and Molecular Dynamics Identify TGF-β2 as a Putative Binding Target of Dioscin. (A) 2D Chemical Structure and MM2-Optimized 3D Model of Dioscin. (B) Molecular docking simulation of Dioscin with TGF-β2 (PDB ID: 6I9J) performed using AutoDock Vina. Left panel shows the overall docking pose of Dioscin within the TGF-β2 binding pocket. Right panel shows detailed interactions with key amino acid residues (ASN405, MET406, CYS350, SER377, SER404, TYR341). The calculated binding energy is −7.9 kcal/mol, suggesting a potential interaction that warrants experimental validation. (C) Root-mean-square deviation (RMSD) profile of the TGFB2 C-alpha atoms as a function of simulation time (ns). (D) Root-mean-square fluctuation (RMSF) profile of TGFB2 amino acid residues during the trajectory.

### 3.7 Dioscin suppresses D-gal-induced activation of the TGF-β2 and PI3K/Akt/β-catenin pathway

Based on our bioinformatic predictions and molecular docking results suggesting TGF-β2 as a computationally predicted potential target, we next investigated whether Dioscin modulates the TGF-β2 signaling axis and its downstream effectors in D-gal-induced RPE cells.

Initially, we assessed the impact of D-gal treatment on the mRNA expression levels of key components within this signaling axis. Our data demonstrated that D-gal treatment led to a marked upregulation of *TGF-β2*, *PI3K*, *Akt*, and *GSK-3β* mRNA levels compared to the control group. Notably, co-treatment with Dioscin effectively reversed these D-gal-induced alterations in mRNA expression, significantly reducing their levels (Fig 7A). We further confirmed this regulatory effect at the protein level through Western blot analysis (Fig 7B). D-gal treatment notably enhanced the phosphorylation of PI3K and Akt (Fig 7C-7D), indicating activation of this pathway. Concurrently, D-gal treatment also resulted in a significant increase in the protein abundance of key signaling components, including both forms of TGF-β2, PKC, GSK-3β, and the critical downstream effector β-catenin. Importantly, the administration of Dioscin effectively normalized these D-gal-induced protein expression and phosphorylation changes, indicating a comprehensive suppressive effect on the entire signaling cascade (Fig 7E-7I).

**Fig 7:**
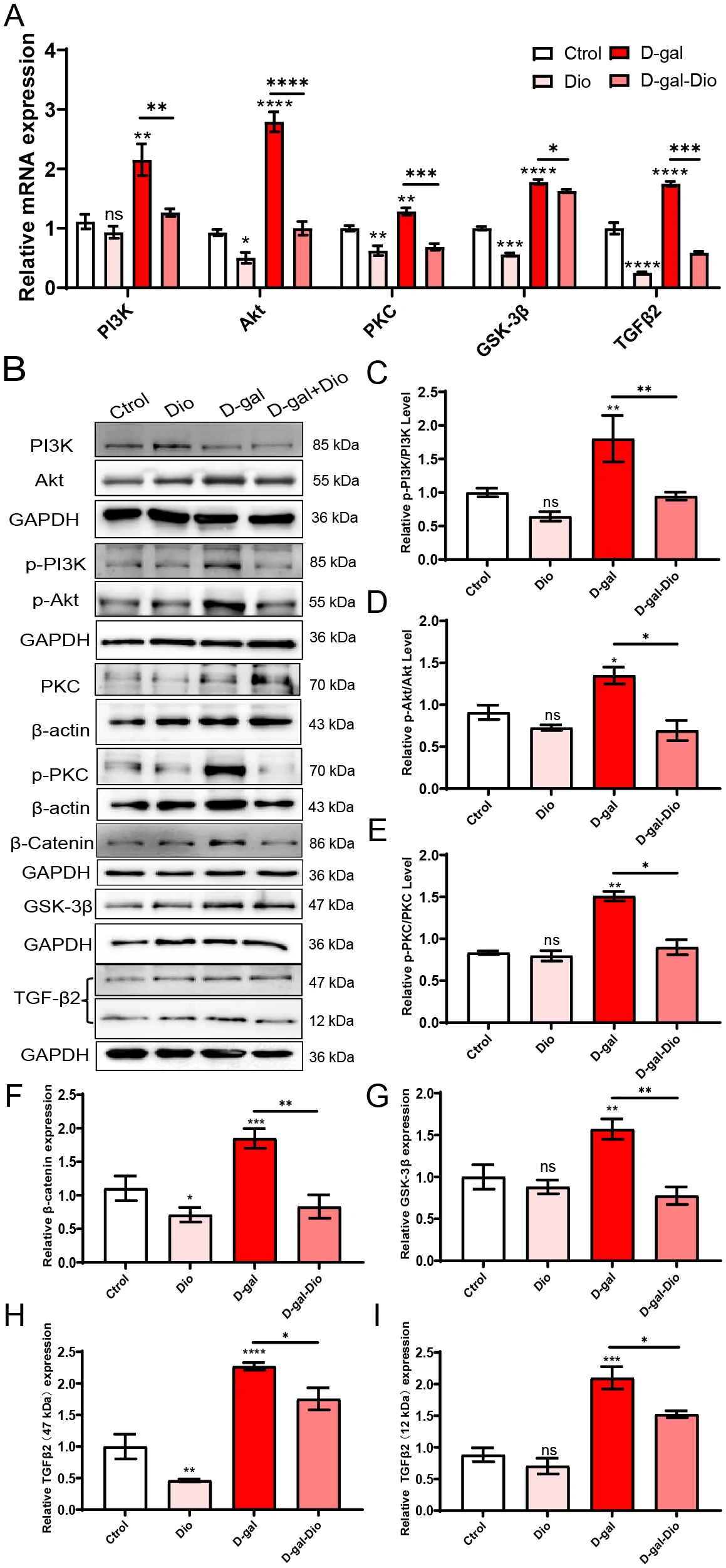
Dioscin suppresses D-gal-induced activation of the PI3K/Akt pathway and TGF-β2 signaling pathway in ARPE-19 cells. (A) Relative mRNA level of *PI3K*, *Akt*, *PKC*, *GSK-3β* and *TGF-β2* in ARPE-19 cells (*n* = 3 independent experiments). (B) Protein levels were determined by Western blot analysis (*n* = 3 independent experiments). Representative Western blot images showing protein levels of key components in the TGF-β2/PI3K/Akt/β-catenin pathway. **(C-I)** Densitometric quantification of protein bands normalized to β-actin (*n* = 3 independent experiments). Data are shown as mean ± S.D. Statistical significance was determined by one-way ANOVA with Tukey’s post-hoc test. *p < 0.05, **p < 0.01, ***p < 0.001, ****p < 0.0001; ns, not significant.

Collectively, these multi-level analyses unequivocally establish that Dioscin acts as a potent inhibitor of the D-gal-induced TGF-β2 and PI3K/Akt/β-catenin axis activation. This finding not only highlights the role of Dioscin in modulating this critical pathogenic pathway but also provides significant molecular evidence for understanding its potential mechanisms in potential therapeutic candidate for AMD.

### 3.8 TGF-β2 expression is elevated in arterial endothelial cells and choriocapillaris endothelial cells of AMD patients

To delineate the cellular sources of TGF-β2 in AMD pathogenesis, we analyzed single-cell transcriptomic data of the macular choroid from both AMD patients and normal controls. Following quality control and clustering, we identified 9 distinct cell types based on their specific marker genes (Fig 8A-8C). The proportion of arterial endothelial cells was significantly elevated in the AMD group (Fig 8D). And we found that TGF-β2 was primarily expressed in Choriocapillaris Endothelial Cells, Arterial Endothelial Cells, Fibroblasts, and Smooth Muscle Cells (Fig 8E). Importantly, compared to the control group, TGF-β2 expression was significantly upregulated specifically in the Choriocapillaris and Arterial Endothelial Cells of AMD patients (Fig 8F-8G). Collectively, these results not only delineate the cellular landscape of the macular choroid but also pinpoint vascular endothelial cells as key sites of TGF-β2 dysregulation in AMD, suggesting that TGF-β2 signaling within these cells may act as a critical pathogenic driver, potentially contributing to choroidal vascular dysfunction and degeneration. Importantly, the specific upregulation of TGF-β2 in choroidal vascular endothelial cells suggests a potential paracrine signaling axis between the choroidal vasculature and the RPE. This indicates that in AMD, pathological signals from the vascular microenvironment may be transmitted to RPE cells via TGF-β2, contributing to the induction of senescence and EMT. This finding provides a cellular basis for understanding the vascular-RPE crosstalk in AMD pathogenesis and establishes a conceptual link between our bioinformatic observations and subsequent in vitro mechanistic studies in RPE cells.

**Fig 8:**
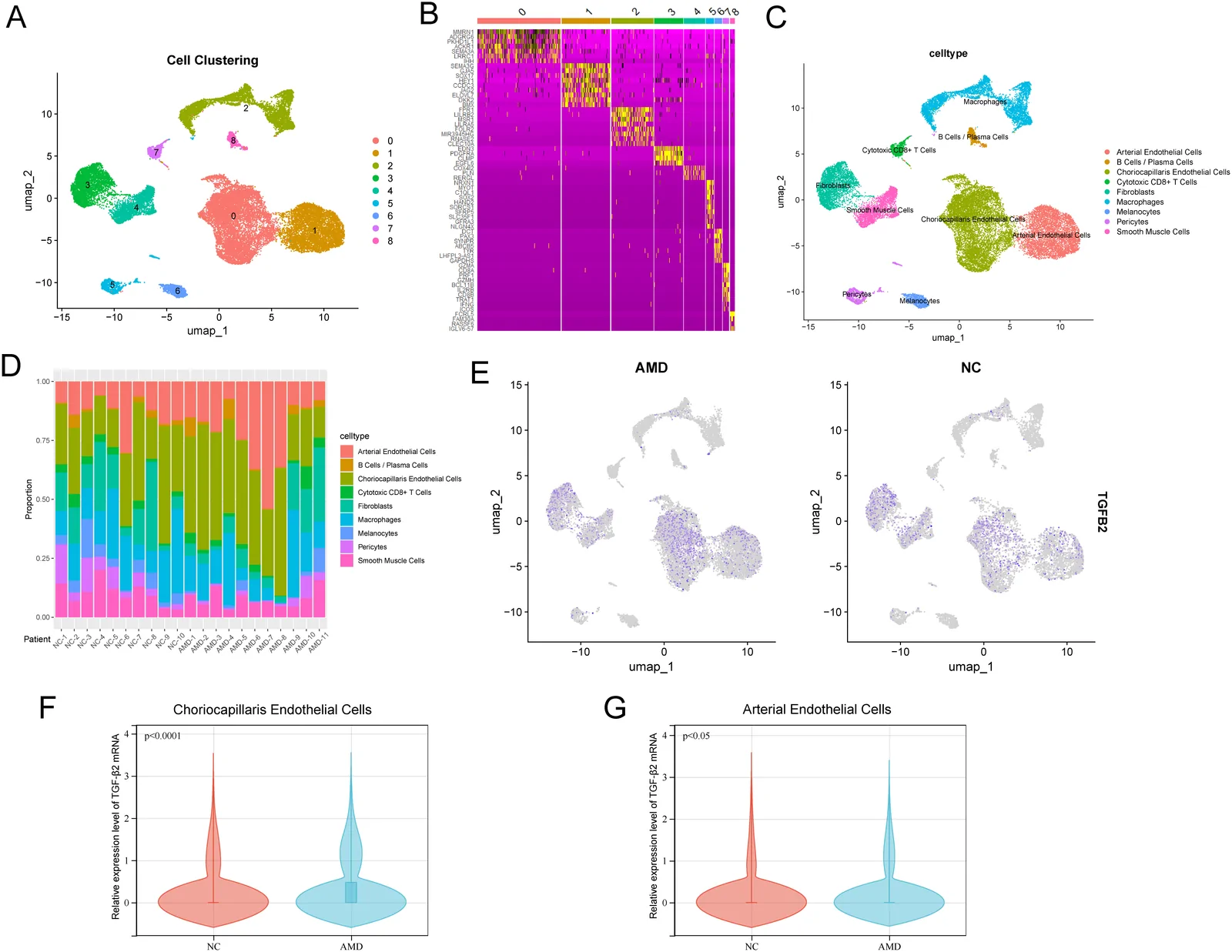
Single-cell transcriptomic analysis reveals TGF-β2 upregulation in choroidal vascular endothelial cells of AMD patients. (A) Cluster analysis identified 9 distinct cell populations. (B) Heatmap showing top marker genes for each cell cluster. (C) UMAP projection of all cells, color-coded by cell type. (D) Relative composition of cell types in normal control (NC) and AMD samples. (E) Feature plot showing TGF-β2 expression distribution across different cell types in the macular choroid. (F-G) TGF-β2 expression in choriocapillaris endothelial and arterial endothelial cells: NC vs AMD (Wilcoxon test).

## 4. Discussion

Age-related macular degeneration (AMD) is a leading cause of irreversible vision loss in the elderly. Retinal pigment epithelium (RPE) dysfunction, particularly cellular senescence and epithelial-mesenchymal transition (EMT), represents a critical early pathological event [38,39]. In this study, we demonstrate that Dioscin, a natural steroidal saponin, exerts protective effects against D-galactose-induced RPE senescence and EMT in both in vivo and in vitro models. Using integrated bioinformatics analysis and molecular docking, we identify transforming growth factor-beta 2 (TGF-β2) as a potential target of Dioscin. Mechanistically, we show that Dioscin suppresses the TGF-β2-mediated PI3K/Akt/β-catenin signaling pathway, thereby attenuating RPE senescence and EMT. Furthermore, single-cell transcriptomic analysis of AMD patient samples reveals that TGF-β2 is specifically upregulated in choroidal vascular endothelial cells, suggesting a paracrine signaling axis between the choroidal vasculature and RPE that may be disrupted by Dioscin.

Dioscin has previously been reported to exert anti‑aging, anti‑inflammatory, and anti‑EMT effects in other systems. Specifically, Dioscin alleviates Alzheimer’s disease via RAGE/NOX4-mediated oxidative stress [18], inhibits tumor stemness and EMT through Akt/GSK3β/β‑catenin signaling in osteosarcoma [40], and suppresses EMT via AKT/GSK3β/mTOR axis in lung adenocarcinoma [41]. Therefore, rather than claiming an entirely novel mechanism, our study extends these known pharmacological actions of Dioscin to an RPE senescence and AMD like context. The specific novel contribution lies in identifying TGF-β2 as a potential vascular derived paracrine mediator.

The D-galactose-induced aging model used in this study does not fully recapitulate the entire pathological spectrum of human AMD, including drusen formation and complement activation [42,43]. Therefore, our model should be interpreted as an AMD-like RPE senescence model rather than a complete surrogate for clinical AMD. Nevertheless, this model effectively captures key features of early dry AMD pathology, including RPE layer disorganization, upregulation of senescence-associated markers (p16, p21, SA-β-gal), and induction of EMT-like phenotypic changes [44]. For in vitro studies, we utilized ARPE-19 cells as an initial screening model. While these cells in subconfluent culture do not fully reflect the polarized state of primary RPE, they represent a widely accepted system for preliminary mechanistic validation [45]. The concordance between in vivo and in vitro results strengthens the validity of our findings.

A key and unexpected finding from our bioinformatic analysis was the cell-type-specific upregulation of TGF-β2. Single-cell transcriptomic data (GSE183320) revealed that TGF-β2 is predominantly expressed in choriocapillaris and arterial endothelial cells, where its level is significantly elevated, rather than in RPE cells themselves. This observation initially appeared to conflict with our in vitro experiments focusing on RPE cells. However, given the anatomical proximity of the choroidal vasculature to the RPE monolayer separated by Bruch’s membrane [46], we propose that elevated TGF-β2 secreted by choroidal endothelial cells may act in a paracrine manner on adjacent RPE cells. This hypothesis is supported by our data showing that exogenous D-gal treatment upregulates TGF-β2 in RPE cells in vitro, and that Dioscin suppresses this upregulation. Thus, while the primary source of pathogenic TGF-β2 in AMD may be vascular endothelial cells, RPE cells can both respond to and, under stress, produce TGF-β2, creating potential feed-forward loops that drive senescence and EMT.

The PI3K/Akt signaling pathway is a central regulator of senescence and EMT [47]. Emerging evidence indicates that TGF-β2 can activate PI3K/Akt signaling to promote EMT in RPE cells [48]. Our results demonstrate that D-gal induction leads to increased phosphorylation of PI3K and Akt, elevated levels of PKC and GSK-3β, and stabilization of β-catenin. The most significant finding is that Dioscin co-treatment potently suppresses the activation of this entire signaling network, reducing both mRNA and protein levels of TGF-β2, PI3K, Akt, PKC, GSK-3β, and active β-catenin. We therefore propose a mechanistic model wherein Dioscin, potentially through modulation of TGF-β2 signaling, inhibits the D-gal-induced activation of the PI3K/Akt/β-catenin axis, thereby alleviating downstream transcriptional programs responsible for cellular senescence and EMT.

Several limitations of this study should be acknowledged. First, the D-galactose-induced aging model does not fully recapitulate human AMD pathology (e.g., drusen, complement activation). Future validation in sodium iodate (NaIO_3_) or genetic AMD models is warranted [49–51]. Second, our in vivo study lacked a positive control and AMD-relevant functional endpoints (OCT, ERG, RPE flat mount). Therefore, our data should be interpreted as evidence of reduced RPE aging-like phenotypes rather than proof of AMD protection. Third, the paracrine signaling hypothesis requires direct testing via co-culture systems or endothelial-specific TGF-β2 knockdown. Fourth, molecular docking and dynamics simulations alone do not prove direct binding; experimental techniques such as SPR, CETSA, or DARTS are needed to confirm that TGF-β2 is a direct target of Dioscin. Fifth, dose-response relationships and ocular pharmacokinetics were not evaluated; future LC-MS/MS studies should measure Dioscin levels in the RPE/choroid. Sixth, the clinical translatability remains unknown, as bioavailability and long-term safety of Dioscin have not been established.

In summary, this study provides evidence that Dioscin counteracts D-gal-induced RPE senescence and EMT through modulation of the TGF-β2/PI3K/Akt/β-catenin axis. The specific upregulation of TGF-β2 in choroidal endothelial cells suggests a vascular-RPE crosstalk mechanism in AMD pathogenesis. While further studies are needed to validate direct target engagement and evaluate efficacy in more clinically relevant models, our findings position Dioscin as a promising natural compound for potential therapeutic candidate for AMD and highlight TGF-β2 as a potential molecular bridge between choroidal pathology and RPE degeneration.

## 5. Conclusion

In conclusion, this study demonstrates that Dioscin alleviates D-galactose-induced RPE senescence and epithelial-mesenchymal transition in both in vivo and in vitro models. Mechanistically, we show that Dioscin suppresses the TGF-β2/PI3K/Akt/β-catenin signaling axis, which is activated under aging-related stress. Integrated bioinformatics analysis (including molecular docking and dynamics simulations) and single-cell transcriptomics further reveal that TGF-β2 is specifically upregulated in choroidal vascular endothelial cells in AMD patients, suggesting a potential paracrine signaling mechanism linking choroidal pathology to RPE dysfunction. In summary, our findings indicate that Dioscin alleviates RPE senescence and EMT in a D‑galactose‑induced AMD‑like model. Further studies in clinically relevant AMD models are warranted to assess its translational potential.

## Supporting information

S1 Table Primer sequences used for RT-qPCR analysis of senescence- and AMD-associated genes in mouse tissues and ARPE-19 cells. (ZIP)

S1 File Raw images. (PDF)

## Abbreviations

AMD: Age-related macular degeneration
Akt: RAC-alpha serine/threonine-protein kinase
Anti-VEGF: Anti-vascular endothelial growth factor
AP-1: Activator Protein-1
AREDS: Age-Related Eye Disease Study
Aβ: Amyloid-Beta
BP: biological processes
CC: cellular components
DAPI: 4’, 6-Diamidino-2-Phenylindole
DEGs: Differentially expressed genes
D-gal: D-galactose
E-cadherin: Epithelial Cadherin
EMT: Epithelial-mesenchymal transition
FBS: Fetal Bovine Serum
GA: Geographic atrophy
GAPDH: Glyceraldehyde-3-Phosphate Dehydrogenase
GEO: Gene Expression Omnibus
GO: Gene Ontology
GPx: Glutathione Peroxidase
GSK-3β: Glycogen Synthase Kinase-3 beta
H&E: Hematoxylin and eosin
HRP: Horseradish Peroxidase
IF: Immunofluorescence
MF: Molecular functions
MI/R: Myocardial ischemia/reperfusion
MMP1: Matrix Metallopeptidase 1
MTT: Methylthiazolyldiphenyl-tetrazolium bromide
N-cadherin: Neural Cadherin
p16: Cyclin-dependent kinase inhibitor 2A
p21: Cyclin-dependent kinase inhibitor 1
PI3K: Phosphatidylinositol 3-kinase
PPI: Protein-Protein Interaction
p-PKC: Phosphorylated-Protein Kinase C
ROS: Reactive Oxygen Species
RPE: Retinal pigment epithelium
RT-qPCR: Real-time quantitative PCR
SA-β-Gal: Senescence-associated-β-galactosidase
SOD: Superoxide Dismutase
TGF-β2: Transforming Growth Factor-beta 2
VITD3: Vitamin D3
β-catenin: Beta-Catenin
SPR: Surface plasmon resonance
CETSA: Cellular thermal shift assay
DARTS: Drug affinity responsive target stability
LC-MS/MS: Liquid Chromatography-Tandem Mass Spectrometry
SPR: Surface Plasmon Resonance
