# Supplementary material for: Dioscin attenuates D‑galactose‑induced retinal pigment epithelium senescence and epithelial–mesenchymal transition via the TGF‑β2/PI3K/Akt/β‑catenin pathway
Source: PLoS One. 2026 Sep 28;21(9):e0359423. doi: 10.1371/journal.pone.0359423 (PMC13618958; doi:10.1371/journal.pone.0359423)
Supplement: Supplementary file 1 [file pone.0359423.s002.pdf]

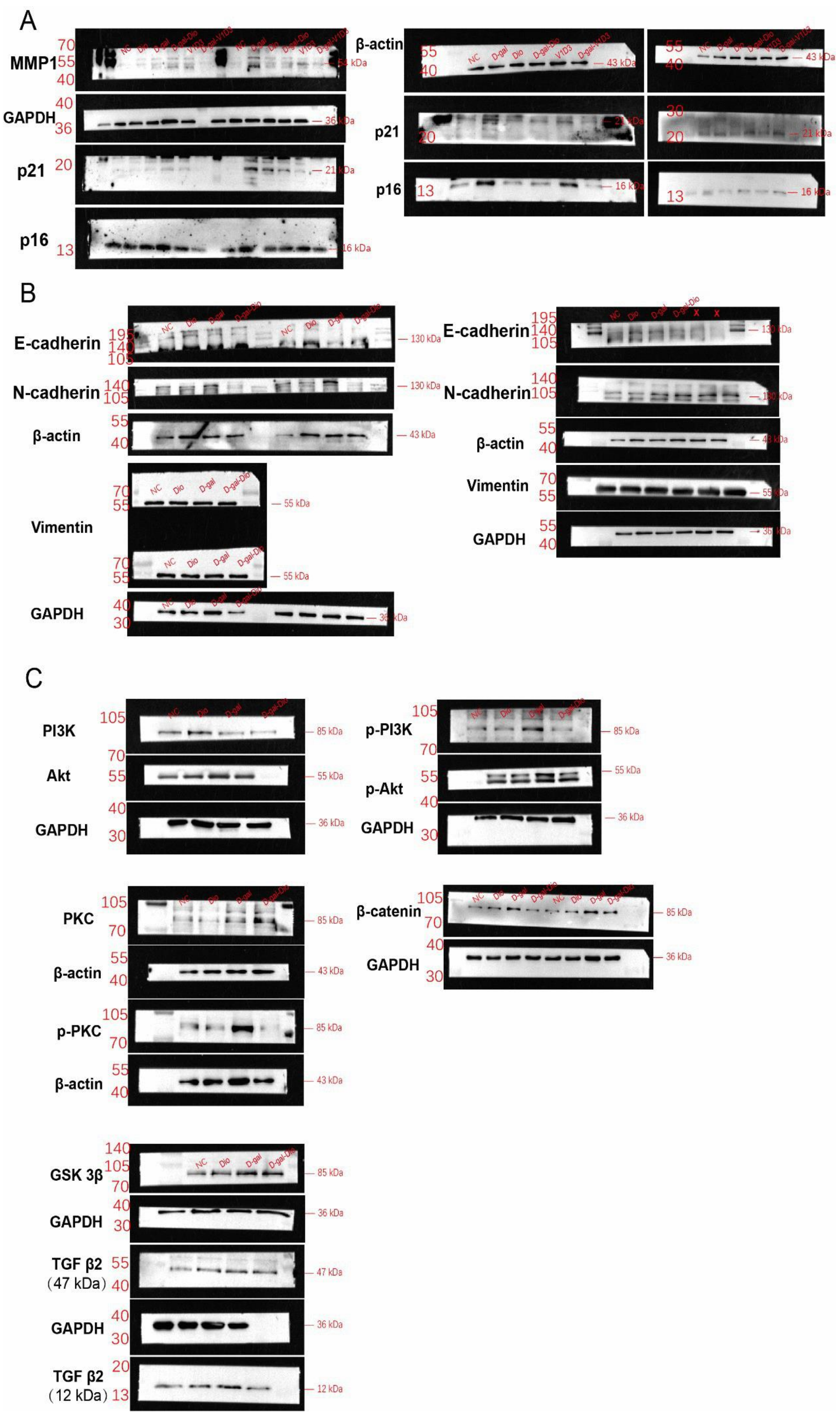
A
MMP1
GAPDH
p21
p16
β-actin
p21
p16
NC
Dio
D-gal
D-gal-Dio
VitD3
D-gal-VitD3
54 kDa
36 kDa
21 kDa
16 kDa
43 kDa
B
E-cadherin
N-cadherin
β-actin
Vimentin
GAPDH
E-cadherin
N-cadherin
β-actin
Vimentin
GAPDH
130 kDa
43 kDa
55 kDa
36 kDa
C
PI3K
Akt
GAPDH
p-PI3K
p-Akt
GAPDH
PKC
β-actin
p-PKC
β-actin
β-catenin
GAPDH
GSK 3β
GAPDH
TGF β2
(47 kDa)
GAPDH
TGF β2
(12 kDa)
85 kDa
55 kDa
36 kDa
43 kDa
47 kDa
12 kDa
