# Supplementary material for: Dioscin attenuates D‑galactose‑induced retinal pigment epithelium senescence and epithelial–mesenchymal transition via the TGF‑β2/PI3K/Akt/β‑catenin pathway
Source: PLoS One. 2026 Sep 28;21(9):e0359423. doi: 10.1371/journal.pone.0359423 (PMC13618958; doi:10.1371/journal.pone.0359423)
Supplement: Supplementary file 2 [file pone.0359423.s001.zip › 新建文件夹/Table S1.docx]

| Mouse-β-actin-F | GGCTGTATTCCCCTCCATCG |
| --- | --- |
| Mouse-β-actin-R | CCAGTTGGTAACAATGCCATGT |
| Mouse-p21-F1 | CGAGAACGGTGGAACTTTGAC |
| Mouse-p21-R1 | CCAGGGCTCAGGTAGACCTT |
| Mouse-p16-F1 | CTCCCGTCTTTGCAGGAATCG |
| Mouse-p16-F1 | CCACCTGAACGTCCATCATC |
| Mouse-N-cadherin-F1 | ACCAGCCTACGAATTGGAGA |
| Mouse-N-cadherin-R1 | GCGTTGTTATCGTCTCCTCG |
| Mouse-E-cadherin-F1 | CTGGGAATCAGATGTTGCCAAC |
| Mouse-E-cadherin-R1 | TGTTGTCCTGCTCGCCTTC |
| Mouse-Vimentin-F1 | CGGCGCAGTCCAACAC |
| Mouse-Vimentin-R1 | ACCAGAGGGAGTGAATCCAGA |
| Human-N-cadherin-F1 | AGCCAACCTTAACTGAGGAGT |
| Human-N-cadherin-R1 | GGCAAGTTGATTGGAGGGATG |
| Human-Vimentin-F1 | AGTCCACTGAGTACCGGAGAC |
| Human-Vimentin-R1 | CATTTCACGCATCTGGCGTTC |
| Human-E-cadherin-F1 | CGAGAGCTACACGTTCACGG |
| Human-E-cadherin-R1 | GGGTGTCGAGGGAAAAATAGG |
| Human-β-actin-F | CACCATTGGCAATGAGCGGTTC |
| Human-β-actin-R | AGGTCTTTGCGGATGTCCACGT |
| Human-TGF-β2-F | CTTGTGCTCCAGACAGTCCC |
| Human-TGF-β2-R | GCTCAATCCGTTGTTCAGGC |
| Human-PKC-F | GTCGAGGGTTTGCCGCT |
| Human-PKC-R | GGGCCAGTTTCAGCTTCAGA |
| Human-AKT-F | GCCCGAAGACGGGAGC |
| Human-AKT-R | CGCTCATGGTGCCCGAG |
| Human-PI3K-F | GACCCGATGCGGTTAGAGC |
| Human-PI3K-R | GCCTCACGGAGGCATTCTAA |

Table S1 RT-qPCR primers for genes associated with cell senescence and AMD in cells or mouse tissues colon mice.
